# Hierarchical dynamic convolutional neural network for laryngeal disease classification

**DOI:** 10.1038/s41598-022-18217-5

**Published:** 2022-08-17

**Authors:** Shaoli Wang, Yingying Chen, Siying Chen, Qionglei Zhong, Kaiyan Zhang

**Affiliations:** Hainan General Hospital (Hainan Affiliated Hospital of Hainan Medical University), Xiuhua road, Hainan, China

**Keywords:** Anatomy, Diseases, Medical research

## Abstract

Laryngeal disease classification is a relatively hard task in medical image processing resulting from its complex structures and varying viewpoints in data collection. Some existing methods try to tackle this task via the convolutional neural network, but they more or less ignore the intrinsic difficulty differences among different input samples and suffer from high training complexity. In order to better resolve these problems, an end-to-end Hierarchical Dynamic Convolutional Network (HDCNet) is proposed, which can dynamically process the input samples based on their difficulty. For the easy-classified samples, the HDCNet processes them with a smaller resolution and a relatively small network, while the difficult samples are passed to a large network with a larger resolution for more accurate classification results. Furthermore, a Feature Reuse Module (FRM) is designed to transfer the features learned by the small network to the corresponding block in the deep network to enhance the overall performance of some rather complicated samples. To validate the effectiveness of the proposed HDCNet, comprehensive experiments are conducted on the public available laryngeal disease classification dataset and HDCNet provides superior performances compared with other current state-of-the-art methods.

## Introduction

Laryngeal disease receives increasing attention as it is the third most prevalent disease only behind colds and coughs and occurs in all ages^1^. According to the China over-the-counter (OTC) Market and Media Research data, over 40% residents have more or less experienced sore throat or other throat diseases in 2009. Laryngoscopy is a primary tool for diagnosing laryngeal disease. However, the diagnosis of the laryngeal disease is rather difficult even for an experienced endoscopist since lesion areas may be easily overlooked or misdiagnosis for the laryngeal images given limited image quality and the complex laryngeal structures^2^, leading to a wrong judgment and even irredeemable damage to patients' health. In addition, the experiences and subjective biases of the endoscopists also affect the diagnosis results^3^. Consequently, the computer-aided-diagnosis (CAD) system for laryngoscopy starts to gain wider popularity in order to aid doctors with more reliable and objective diagnosis results. Moreover, such a system is able to reduce the tremendous and cumbersome workload of endoscopist which significantly improve the diagnostic efficiency^4^.

Early methods for CAD include three stages: hand-craft feature extraction, feature selection, and classification^5^. These methods highly rely on the expertise of experienced experts and also are limited in performance. More recently, inspired by the prosperity of deep learning methods in the general image classification task, the researcher of medical imaging communities have focused on designing end-to-end deep learning based methods to tackle the laryngeal image classification task. With resort powerful representation capacity, deep learning based methods exhibit astonishing performance and have dominated this field^2^.

For example, Xiong et al.^2^ propose a deep convolutional neural network (DCNN) for automatically detecting laryngeal cancer in laryngeal images. They train the network in a transfer learning scheme by initializing the network with the weights pre-trained on ImageNet^7^ to absorb extra information from external data for boosting the model performance over the laryngeal data. Many other works also adopt the DCNN and the transfer learning scheme for laryngeal cancer classification^8,9^. Cho et al.^3^ conduct comparisons among four DCNNs on the task of common nine laryngeal diseases classification. According to their experiments, all trained DCNNs exhibit extraordinary performance, even better than visual assessments of human beings in discriminating seven of nine diseases. Recently, Yin et al.^1^ build a new public laryngeal image dataset named Laryngoscope8. They further propose a three-stage method and reach state-of-the-art performance in their proposed dataset. Despite the huge success of the above-mentioned methods, they more or less suffer from two major defects:

(1) All images are processed by a single DCNN which ignores the differences in difficulty between input samples.

(2) They often need heavy computational costs. For instance, the method employed by^1^ requires three different training networks.

To solve these problems, we proposed a Hierarchical Dynamic Convolutional network (HDCNet) which not only dynamically processes input samples according to their classification difficulty, but also avoids the heavy computational overhead. In particular, the HDCNet adopts a dynamic two-stream network to process different samples with different paths according to the difficulty of input samples. Moreover, a Feature Reuse Module (FRM) is designed to incorporate preliminary features from the small stream into the large stream to further improve classification accuracy. We conduct experiments on the Laryngoscope8, a public laryngeal disease classification dataset, and HDCNet achieves the best result compared with all existing competitors. In summary, this letter has two contributions:

1. A novel HDCNet is proposed to solve laryngeal image classification in a more effective way.
2. The proposed HDCNet achieves higher performance compared with current SOTA methods.

## Methods

**Figure 1:**
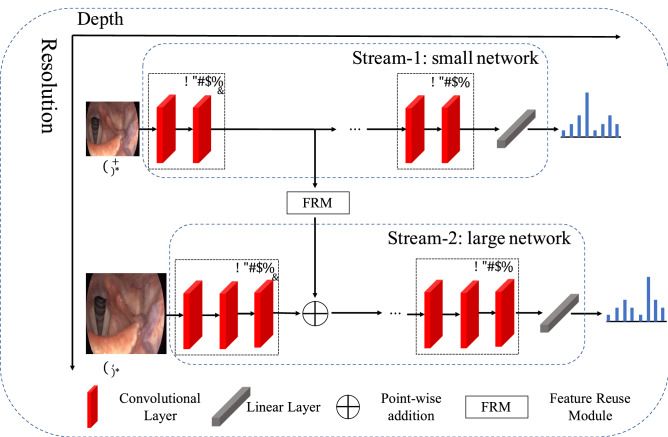
The overall pipeline of the proposed HDCNet.

To better diagnose the laryngeal diseases from the input samples, a Hierarchical Dynamic Convolutional Network (HDCNet) is proposed in an upstream manner. As shown in Fig. 1, the proposed HDCNet consists of two parts: (1) A dynamic two-stream network to process different samples with different computational graphs; (2) A Feature Reuse Module (FRM) to incorporate preliminary features from the small stream into the large stream for further enhancement in classification results. In the rest of this section, more details on these two parts are given to better illustrate the whole training and inference process.

### Dynamic two-stream network

Previous methods only employ a single DCNN for all input samples. Suppose the input samples ${X}_{in}\in {\mathbb{R}}^{C\times H\times W}$, where $C$ denotes the number of input channels, $H$ and $W$ are the width and height, respectively. Conventional DCNNs usually contain several consecutive blocks $bloc{k}_{i}$, each of which consists of several convolutional layers. Then, the prediction of the whole network with respect to the input samples can be denoted as,

$$p = softmax\left( {f\left( {block_{i} \odot block_{i - 1} \odot \cdots \odot block_{1} \left( {X_{in} } \right)} \right)} \right),$$

where the $\odot$ denotes applying the convolutional block $bloc{k}_{i}$ to its input, $f(.)$ denotes the linear layer and $softmax$ is the softmax function that normalizes its input into a probability distribution. $p\in {\mathbb{R}}^{n\times 1}$ is the output possibility distribution, where ${p}_{i}$ denotes the possibility for ${i}_{th}$ disease, and $n$ is the number of diseases. Although the conventional neural networks reach record-breaking performance in laryngeal disease classification, it ignores the intrinsic difficulty differences among input samples. For an easy-classified sample, a straightforward network with fewer convolutional layers is enough to make an accurate prediction, while the network needs to be deeper and wider in order to classify those hard-classified samples with ambiguous features. To overcome this problem, we design a two-stream classification network in an upstream manner.

As shown in Fig. 1, different from previous methods, the proposed HDCNet has two streams to process the input samples dynamically based on their difficulties. In the proposed HDCNet, all input samples are passed to a small network (i.e., the small stream) with a small input resolution first, whose convolutional block $bloc{k}_{i}$ contains fewer convolutional layers. The small stream can be viewed as the preliminary processing of the input samples, which can filter out easy-diagnosed samples and keep those difficult samples for further classification. Specifically, if the maximum of the output possibility $p$ is larger than a threshold $\tau$ the classification result is believed to be valid enough and does not need to be further processed. As for those samples whose maximum possibility is smaller than the threshold $\tau$, their classification results are believed to be invalid. Thus, a deeper and wider network (i.e., the large stream) that has more convolutional layers with more channels in each convolutional block $bloc{k}_{i}$ is adopted to process them. The input resolution of each sample also increases correspondingly to provide richer details for better classification performance.

By adopting the HDCNet, the intrinsic difficulty of input sample are taken into consideration, where the easy-classified samples can be processed with a small network and the hard-classified samples can be tacked by a larger network. Consequently, HDCNet is able to reduce the computational cost while reaching a higher overall performance. In order to leverage the knowledge from the small network, a Feature Reuse Module (FRM) is employed between each block of the small network and the corresponding block of the large network where the preliminary features learned by the small network are incorporated into the large network. More details of the FRM are given in the next section.

### Feature reuse module

**Figure 2:**
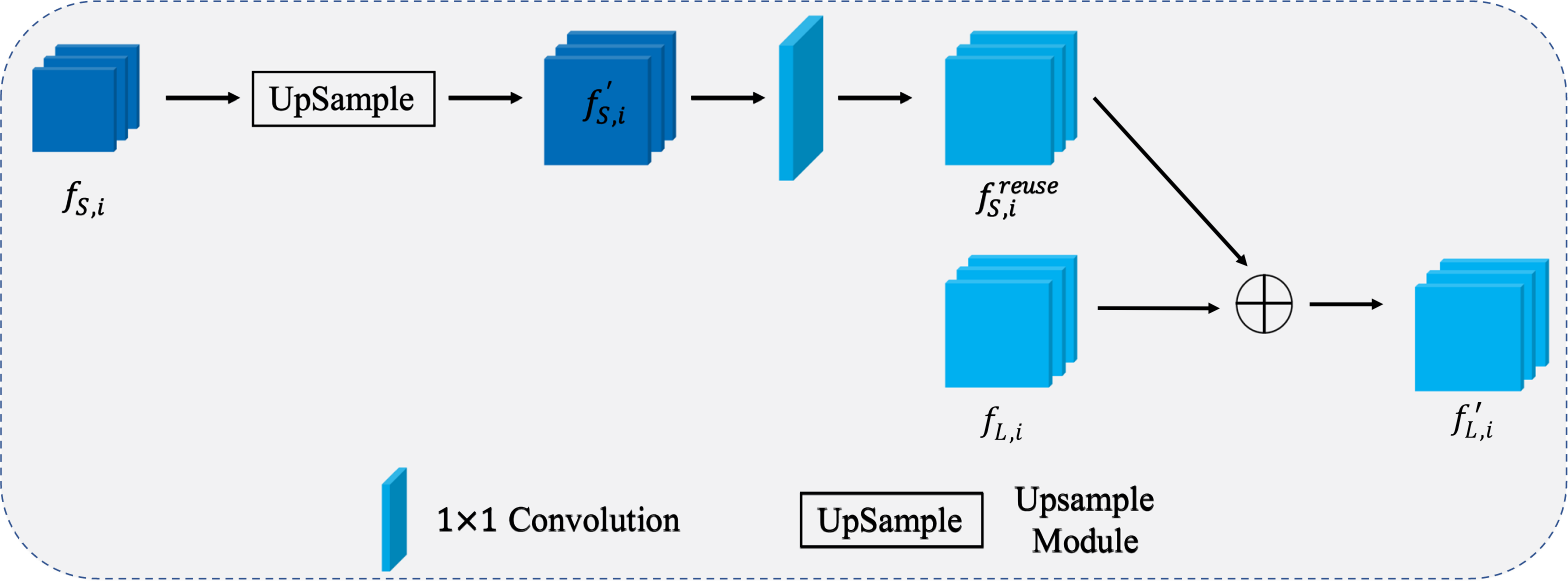
The overall pipeline of the proposed Feature Reuse Module.

Although the small stream of the proposed HDCNet can not provide reliable predictions for difficult samples, it still generates useful features which can be integrated into the large stream to help with further classification. It is worth noting that, to make a better feature transfer between the two streams, we ensure they have the identical number of blocks to make the second can easily use the Feature Reuse Module (FRM). The FRM is plugged between the blocks with the same index, e.g., $bloc{k}_{i}$ in the small stream and the large stream. The structure is presented in Fig. 2.

Denote ${f}_{S,i}\in {\mathbb{R}}^{C\times H\times W}$ as the output feature map from the $bloc{k}_{i}$ of the small stream, it first is passed a upsample module to match the resolution of the feature map of the large stream. The output of this upsample module is denoted as ${f}_{S,i}^{^{\prime}}\in {\mathbb{R}}^{C\times {H}^{^{\prime}}\times {W}^{^{\prime}}}$, where ${H}^{^{\prime}}$ and ${W}^{^{\prime}}$ are the scaled width and height respectively. Then, an $1\times 1$ convolutional layer is used to accomplish feature domain alignment since the features generated by a different network may belong to different feature domains. The computation process of the $1\times 1$ convolutional layer is defined as:

$${f}_{S,i}^{reuse}=Con{v}_{1\times 1}\left({f}_{S,i}^{^{\prime}}\right).$$

Finally, ${f}_{S,i}^{reuse}\in {\mathbb{R}}^{C\times {H}^{^{\prime}}\times {W}^{^{\prime}}}$ is incorporated into the large stream via the point-wise addition. Denote ${f}_{L,i}\in {\mathbb{R}}^{C\times {H}^{^{\prime}}\times {W}^{^{\prime}}}$ as the output feature map from the $bloc{k}_{i}$ of the large stream, $\oplus$ as the point-wise addition operation. The computation process is defined as:

$$f_{L,i}^{^{\prime}} = f_{L,i}^{{}} \oplus f_{S,i}^{reuse} .$$

The ${f}_{L,i}^{^{\prime}}$ is then used as the input of the next block (i.e., $bloc{k}_{i+1}$) in the larger network.

## Experiments

### Experimental setting

#### Datasets

In this letter, Laryngoscope8, a publicly available dataset of laryngeal disease classification proposed by Yin et al.^1^, is adopted to evaluate the proposed HDCNet with current SOTA methods. The dataset contains 3057 images of 1950 patients. A total of 8 distinct labels (Edema, Cancer, Granuloma, Normal, Leukoplakia, Cyst, Nodules, Polyps) are given to each input sample to demonstrate its corresponding disease. The laryngeal images were taken by two laryngoscope devices: Xion Matrix HD3 and Delon HD380B. The input images are preprocessed with several image enhancement methods, including horizontal flips, vertical flips, random cropping and image normalization. The input of the ResNet18 is 224 pixels in width and 224 pixels in height, following the settings in work^1^, while the resolution of the input for the ResNet34 is $336\times 336$ since we find a larger resolution will not bring a convincing increasement in its performance compared to the higher computational cost it brings.

Following Yin et al., we use 70% images as the training samples while the rest are regarded as the test samples to evaluate the performance of compared methods.

#### Evaluation metrics

In particular, the AUC (area under the curve) of each category, the average AUC of all categories, and the overall classification accuracy are adopted as the evaluation metrics to demonstrate the outstanding performance of the HDCNet. The accuracy is defined as:

$$\mathrm{Accuracy }=\frac{\mathrm{number \; of \; correct \; predicted \; samples}}{\mathrm{number \; of \; samples}}.$$

The AUC (area under the curve) measures the area under the Receiver Operating Characteristic (ROC) curve. And the ROC curve can be obtained by plotting the true positive rate (TPR) against the false positive rate (FPR) at all classification thresholds. The average AUC is the average value of the AUC for each category.

#### Implementation details

The proposed HDCNet is trained and evaluated on a single NVIDIA GeForce 1080ti GPU. ResNet18^6^ and ResNet34 are adopted as the small stream and the large stream of the HDCNet, respectively. Note that both ResNet18 and ResNet34 have an identical number of blocks so that the FRM is easy to apply.

The training process includes two steps. In the first step, the two networks (i.e. two streams of the HDCNet) are trained independently for a total of 300 epochs. The input image resolution for the small stream (ResNet18) is set to be $224\times 224$ and a larger image resolution, $336\times 336$, is used in the large stream since the images with a larger resolution are able to retain more details that are vital for better classification accuracy. After that, a small stream (ResNet18) and a large stream (ResNet34) are obtained. In the second step, the large stream (ResNet34) is fine-tuned with the FRM for 50 epochs to achieve better performance.

The learning rate of these two streams is set to be 0.0001 and the Adam optimizer is used to train the HDCNet. The batch size is set to be 16 and the threshold $\tau$ is set to be 0.8.

### Laryngeal images classification results

The comprehensive comparisons are presented in Table 1. As can be seen, the performance of the HDCNet outperforms current SOTA methods in the average AUC of all categories and the overall classification accuracy. Specifically, the average AUC of our HDCNet is 0.910, which is 0.017 higher than that of the previous SOTA method (i.e., Yin et al.)^1^. As for the AUC of each category, our HDCNet obtains the best results in five of eight categories. The largest performance gain (0.043) occurs when classifying the ``Normal'' category. Moreover, the overall classification accuracy of the proposed HDCNet achieves 2.27% performance gain (75.27% VS 73%) when compared with Yin et al..

**Table 1:** The performance evaluation of different methods on the AUC and overall accuracy among all classes, the best results are highlighted in bold.

Methods	Edema	Cancer	Granuloma	Normal	Leukoplakia	Cyst	Nodules	Polyps	average AUC	Accuracy
CheXNet^10^	0.798	0.822	0.979	0.900	0.876	0.685	0.825	0.853	0.843	71%
AG-CNN^11^	0.805	0.879	0.972	0.895	0.896	0.658	0.828	0.838	0.847	71%
Xiong et al.^2^	0.857	0.866	0.978	0.911	0.894	0.705	0.857	0.886	0.870	71%
Yin et al	0.900	0.936	0.965	0.878	0.853	0.849	0.886	0.871	0.893	73%
HDCNet	**0.908**	0.921	0.953	**0.921**	**0.930**	0.835	**0.908**	**0.905**	**0.910**	**75.27%**

It is worth noting that in the method of Yin et al., the input images are first processed by a localization model, i.e., Faster RCNN^12^, to find their critical regions. These critical regions are then sent to a classification model to obtain predicted labels of input images. In contrast to their method, HDCNet performs the whole classification in a uniform framework, which is more efficient.

### Ablation study

#### Effectiveness of components

**Figure 3:**
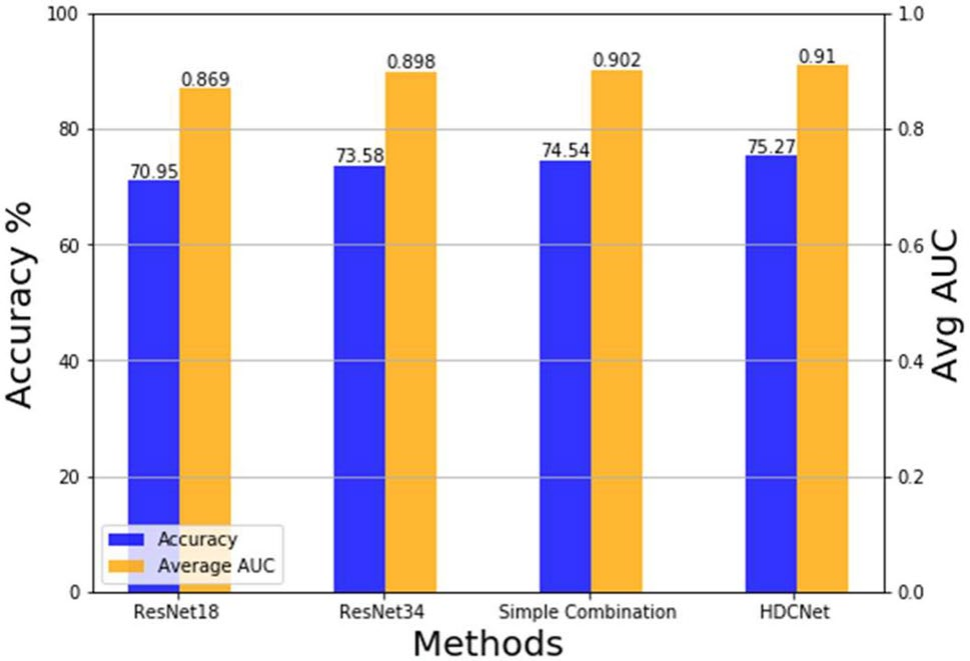
Comparison between different methods.

In Fig. 3, the effectiveness of the proposed components is evaluated. In particular, a single ResNet18 network provides 70.95% accuracy and 0.869 average AUC. It can not achieve satisfactory classification accuracy since the small network is lack representative capacity to label the input images correctly. On the contrary, with a higher representative capacity, the large network, i.e., ResNet34, achieves better accuracy than ResNet18 (73.58% vs 70.95%). Nevertheless, the large network is over-complex and may perform poorly on some simple images resulting from the over-fitting problem. As a result, by simply combining these two networks, i.e., ''simple combination'', the performance increases from 73.58 to 74.54%. The cascading of the two networks provides better performance than applying ResNet18 and ResNet34 solely. However, the simple cascading of these two networks ignores the useful knowledge learned by the small network. Thus, by further applying the FRM to transfer the knowledge from the small network to the large network, i.e., the proposed HDCNet, higher classification accuracy, and average AUC can be achieved. To be more specific, the accuracy increases from 74.54 to 75.27% and the average AUC increases from 0.902 to 0.910 compared with the simple combination.

#### Efficiency of the HDCNet

To demonstrate the efficiency of the proposed HDCNet, we provide the average FLOPs (floating-point per seconds) of different methods with their inference accuracy as following.

As shown in Table 2, the HDCNet realize a better balance between the classification accuracy and computational cost. Although MobileNetv3 and EfficientNetb0 has fewer FLOPs, the performance degrades abruptly compared with other network structures. Furthermore, the purposed HDCNet reaches a rather convincing classification accuracy, i.e. 75.27%, using only 1.73G FLOPs. Using a larger network to substitute the combination of ResNet18 and ResNet34 doesn’t show obvious enhancement in the accuracy regarding the higher FLOPs they acquire.

**Table 2:** Comparison of different methods with the proposed HDCNet according to the Accuracy and FLOPs.

	Model (resolution)	Accuracy (FLOPs)
Single model	MobileNetv3_large_w1 (224)	52.52% (0.12G)
	EfficientNet_b0 (224)	54.17% (0.21G)
	ResNet18 (224)	70.95% (0.91G)
	ResNet34 (336)	73.58% (4.21G)
	ResNet50 (336)	74.15% (4.73G)
HDCNet (combination)	ResNet18 (224) + ResNet34 (336)	75.27% (1.74G)
	ResNet18 (224) + ResNet50 (336)	75.57% (1.87G)
	ResNet34 (224) + ResNet50 (336)	75.90% (2.56G)

#### Visualization of examples

**Figure 4:**
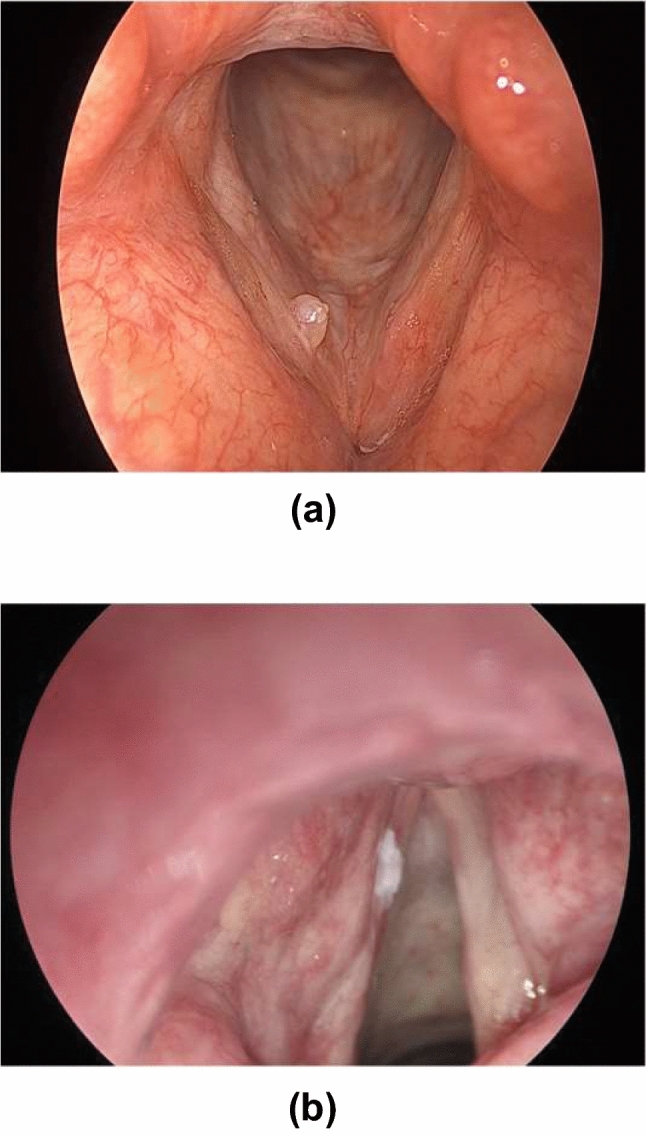
(**a**) and (**b**) are input images that cannot be classified correctly by ResNet18 but can be correctly diagnosed by the proposed HDCNet.

To demonstrate the effectiveness of the proposed HDCNet, we provide examples that can not be classified appropriately in the solo network but are correctly classified by the proposed HDCNet. As shown in Fig. 4, the lesions in these two input images are relatively small and thus hard to identify by the solo network that possesses only limited representative capacity. However, the proposed HDCNet is able to correctly predict their categories.

**Figure 5:**
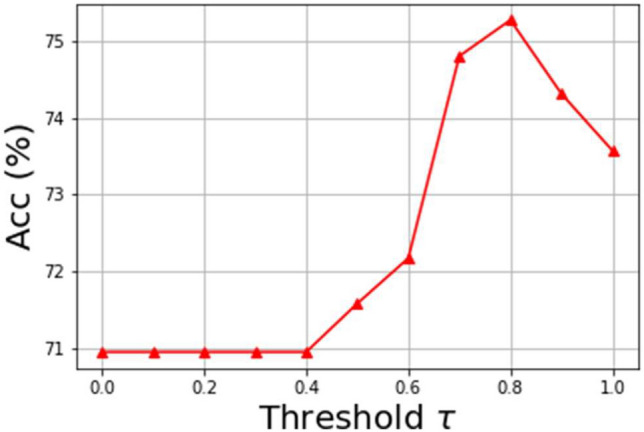
The performance with respect to different thresholds $\tau$. The accuracy is used as the metric for selecting the best $\tau$.

Selection of the threshold $\tau$. The threshold $\tau$ is used to determine whether a sample is only classified by the small network or needs further processing by the large network in the HDCNet. Normally speaking, a smaller $\tau$ means more samples are only fed to the small network while a large $\tau$ means more samples are fed to the large network for more accurate classification. The experiments of different $\tau$ are shown in Fig. 5. As can be seen, the highest performance of the HDCNet is obtained when $\tau$ is selected to be 0.8.

## Conclusion

In this letter, we have proposed a novel framework, named Hierarchical Dynamic Convolutional Network (HDCNet), to accomplish the Laryngeal disease classification task. By cascading two different networks with several Feature Reuse Modules, HDCNet classifies different samples dynamically based on their difficulties. The easy-classified samples are classified by a small network with a small resolution, while samples, which cannot be predicted accurately, are passed to a large network for better classification results. The proposed HDCNet has surpassed current SOTA methods with a large margin on the public Laryngoscope8 dataset. In the future, a more dedicated Hierarchical network is intended to be proposed to reach higher classification accuracy on the Laryngeal disease classification task.

## Data Availability

All data generated or analysed during this study are included in the published article^1^ [Web link: https://github.com/greenyin/Laryngoscope8].

## References

[1] Yin L, Liu Y, Pei M, Li J, Wu M, Jia Y (2021). Laryngoscope8: Laryngeal image dataset and classification of laryngeal disease based on attention mechanism. Pattern Recogn. Lett..

[2] Xiong H, Lin P, Yu J-G, Ye J, Xiao L, Tao Y, Jiang Z, Lin W, Liu M, Xu J (2019). Computer-aided diagnosis of laryngeal cancer via deep learning based on laryngoscopic images. EBioMedicine.

[3] Cho WK, Lee YJ, Joo HA, Jeong IS, Choi Y, Nam SY, Kim SY, Choi S-H (2021). Diagnostic accuracies of laryngeal diseases using a convolutional neural network-based image classification system. Laryngoscope.

[4] Luan, B., Sun, Y., Tong, C., Liu, Y., & Liu, H. R-fcn based laryngeal lesion detection. In: *2019 12th International Symposium on Computational Intelligence and Design* (ISCID), vol. 2, pp. 128–131 (2019). IEEE

[5] Miranda, E., Aryuni, M., & Irwansyah, E. A survey of medical image classification techniques. In: 2016 International Conference on Information Management and Technology (ICIMTech), pp. 56–61 (2016). IEEE.

[6] He, K., Zhang, X., Ren, S., & Sun, J.: Deep residual learning for image recognition. In: *Proceedings of the IEEE Conference on Computer Vision and Pattern Recognition*, pp. 770–778 (2016).

[7] Deng, J., Dong, W., Socher, R., Li, L.-J., Li, K., & Fei-Fei, L. Imagenet: A large-scale hierarchical image database. In: *2009 IEEE Conference on Computer Vision and Pattern Recognition*, pp. 248–255 (2009). IEEE.

[8] Esmaeili N, Sharaf E, Gomes Ataide EJ, Illanes A, Boese A, Davaris N, Arens C, Navab N, Friebe M (2021). Deep convolution neural network for laryngeal cancer classification on contact endoscopy-narrow band imaging. Sensors.

[9] He, Y., Cheng, Y., Huang, Z., Xu, W., Hu, R., Cheng, L., He, S., Yue, C., Qin, G., & Wang, Y., et al. A deep convolutional neural network-based method for laryngeal squamous cell carcinoma diagnosis. *Ann. Transl. Med.* **9**(24) (2021). 10.21037/atm-21-6458 PMC8756237 35071491

[10] Rajpurkar, P., Irvin, J., Zhu, K., Yang, B., Mehta, H., Duan, T., Ding, D., Bagul, A., Langlotz, C., & Shpanskaya, K., et al.: Chexnet: Radiologist-level pneumonia detection on chest x-rays with deep learning. arXiv preprint arXiv:1711.05225 (2017).

[11] Guan Q, Huang Y, Zhong Z, Zheng Z, Zheng L, Yang Y (2020). Thorax disease classification with attention guided convolutional neural network. Pattern Recogn. Lett..

[12] Ren, S., He, K., Girshick, R., & Sun, J.: Faster r-cnn: Towards real-time object detection with region proposal networks. *Adv. Neural Inf. Process. Syst.* **28** (2015). 10.1109/TPAMI.2016.2577031 27295650

